# Stylohyoid Complex Syndrome Associated With Unilateral Vocal Cord Palsy: A Case Report

**DOI:** 10.7759/cureus.21666

**Published:** 2022-01-27

**Authors:** Anna Loroch, Sabih Nadeem Qamar, Mehaab Jaffer, Ian Smillie

**Affiliations:** 1 Department of ENT, NHS Greater Glasgow and Clyde, Glasgow, GBR; 2 Department of ENT Surgery, University Hospital Monklands, Airdrie, GBR

**Keywords:** unilateral neck pain, vocal cord paralysis, stylohyoid complex, styalgia, eagle's syndrome

## Abstract

Eagle’s syndrome is a rare cause of cervicofacial pain and is due to abnormalities in the stylohyoid process, stylohyoid ligament or lesser cornu of the hyoid bone. Generally, patients affected by Eagle’s syndrome present with pain in the lateral or upper neck, angle of the mandible, submandibular space and throat (exacerbated by head movements and/or mastication); foreign body sensation; headache and referred otalgia.

A 66-year old gentleman presented with a 36-month history of recurrent pain localising mainly to the right angle of the mandible and radiating to the submandibular triangle. No pathological changes were noted on multiple ultrasound scans. Flexible nasendoscopy revealed a right vocal cord palsy. Initially, the CT scan revealed an abnormality in the stylohyoid complex, and the patient was managed conservatively. Subsequent three-dimensional CT scan noted significant worsening of the abnormality in the stylohyoid complex.

Due to progressive nature of the patient's symptoms and progression of stylohyoid complex calcification noted on imaging, the patient was listed for surgery. He underwent partial styloidectomy and vocal cord injection for cord paralysis secondary to impingement on the vagal nerve by the stylohyoid complex. The patient recovered well and denies any ongoing stylalgia.

Various cases of Eagle’s syndrome have been managed successfully in a conservative manner. However, the authors of this case report suggest that patients with Eagle’s syndrome should be monitored closely. A delay in surgical intervention can lead to complications such as complete ossification of the stylohyoid complex and impingement on surrounding structures. This, in turn, increases intra-operative complexity.

## Introduction

Eagle’s syndrome is caused by pathology in the stylohyoid complex. A new diagnostic classification of this entity was reported by Colby and Del Gaudio as the stylohyoid complex syndrome (SHCS). This encompasses pain caused by abnormalities in either the stylohyoid process, stylohyoid ligament or lesser cornu of the hyoid bone [1].

Abnormal angulation of the styloid process was also deemed to be responsible for stylalgia [2]. Symptoms may vary between patients, including pain in the lateral or upper neck, angle of the mandible, submandibular space and throat (exacerbated by head movements and/or mastication); foreign body sensation in the pharynx; headache and referred otalgia [1,3]. Moreover, symptoms resulting from compression of the abnormal stylohyoid complex can lead to compression of surrounding neurovascular structures. The styloid process should measure less than 3 cm; hence, any elongation noted on imaging could be responsible for the SHCS [2,3].

Herein, we present a case of recurrent neck pain caused by an elongated styloid process and severely ossified stylohyoid complex associated with a unilateral vocal cord (VC) palsy. To our knowledge, this is the first case of VC palsy associated with Eagle’s syndrome.

## Case presentation

A 66-year-old gentleman was referred to an ear, nose and throat (ENT) clinic at University Hospital Monklands with right-sided neck pain. The patient reported a 36-month history of recurrent pain in the right angle of the mandible radiating to the submandibular triangle. He also complained of persistent hoarseness (lasting for a few months) but denied any difficulty breathing. The patient’s past medical history included osteoarthritis, hypertension, type 2 diabetes mellitus (diet controlled), obesity, previous bilateral total hip replacement and knee replacement. Initial clinical examination did not reveal any lumps within the neck, and the right submandibular gland was palpable, non-tender and soft. No abnormalities were noted on contralateral examination. Bimanual palpation of the right lingual sulcus revealed a bony structure measuring 1cm located posteriorly to the mandible. Flexible nasendoscopy (FNE) revealed a unilateral right VC palsy with a bowing effect of the right VC; however, mucosal lesions were seen.

**Figure 1:**
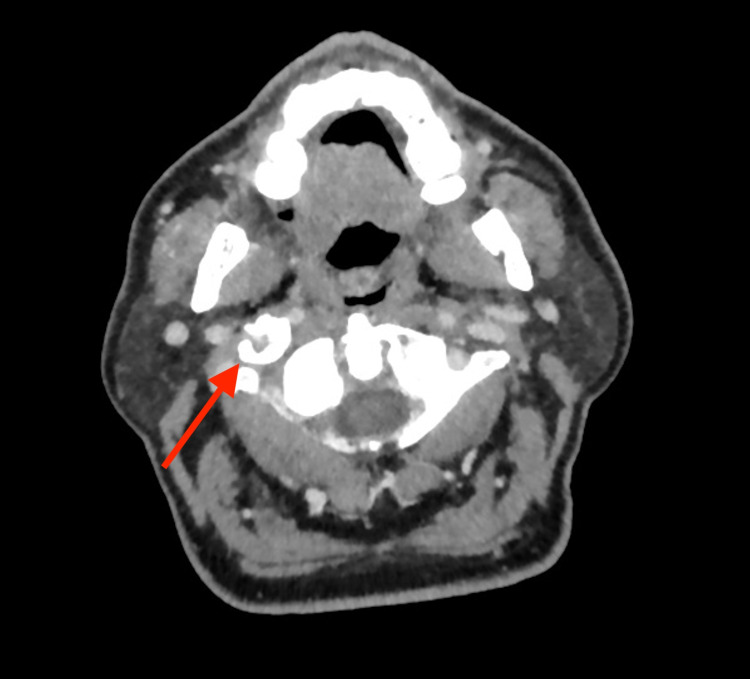
CT of the neck with contrast (axial plane) revealing a long, thick right styloid process (red arrow) consistent with a clinical diagnosis of Eagle's syndrome.

An ultrasound scan of the neck was performed; however, no focal lesions were noted in the thyroid gland and both submandibular glands appeared unremarkable. This was followed by a CT scan to assess the right stylohyoid complex, which showed an elongated, thick right styloid process (measuring 10 cm), extending to the level of the hyoid bone. The appearance was consistent with Eagle’s syndrome (Figure 1).

**Figure 2:**
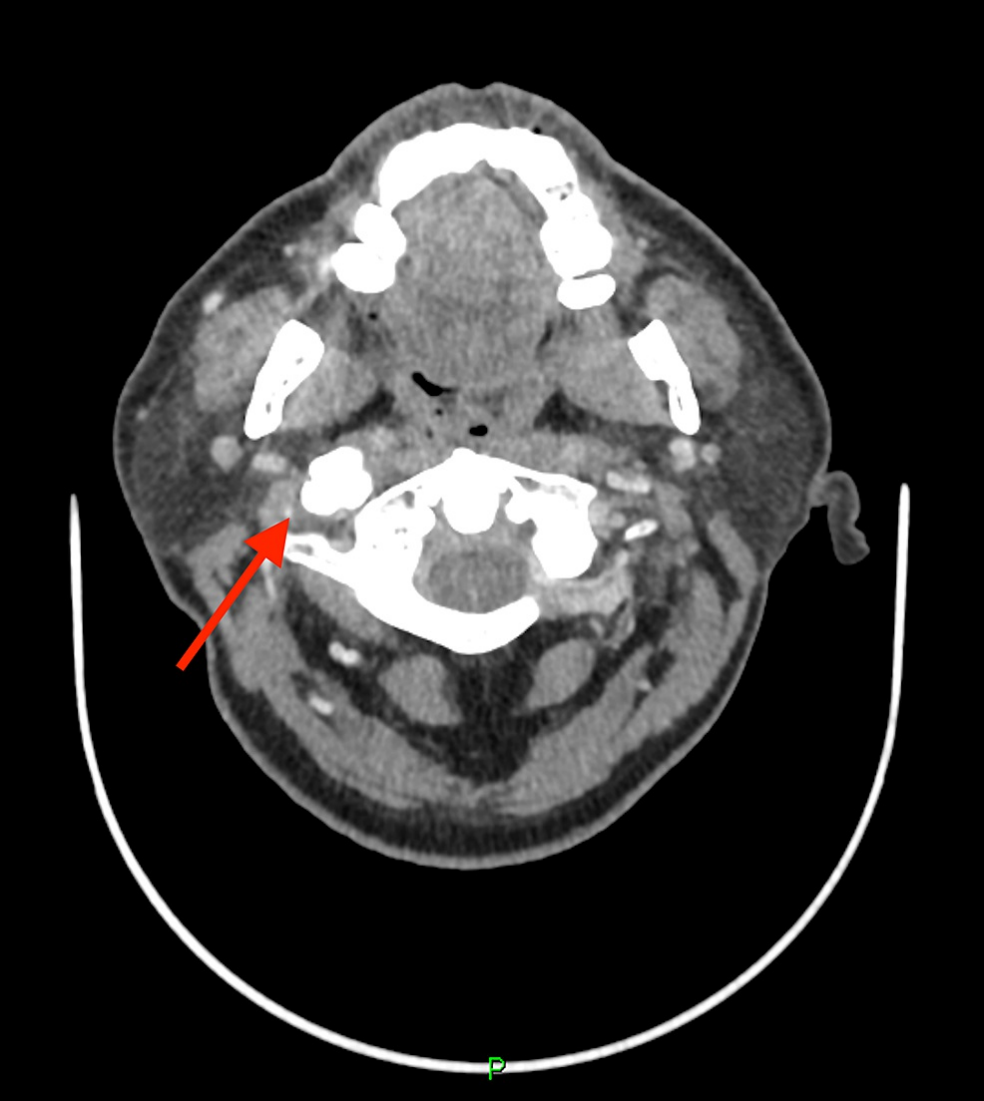
Follow-up CT of the neck with contrast (axial plane) showing calcification of the elongated right stylohyoid ligament (red arrow).

**Figure 3:**
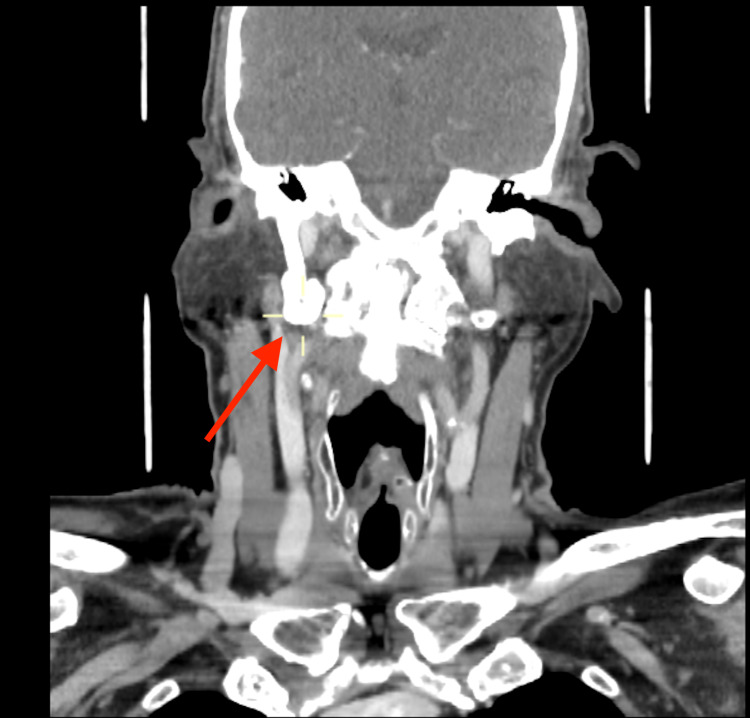
Follow-up CT of the neck and thorax with contrast (coronal plane), showing complete ossification of the stylohyoid ligament and significant bony overgrowth (red arrow).

A conservative management was initially suggested for this patient. During a follow-up review, 13 months later, he reported an improvement in pain with regular analgesia. We offered speech and language assessment of patient’s symptoms caused by right VC fixation; however, the patient declined. A two-year follow-up CT scan revealed marked progression in the patient’s disease. The CT scan noted calcification of the elongated right stylohyoid ligament, marked degeneration of the cervical spine, cervical kyphosis and multilevel bridging osteophytes with minor anterior slip by 2 mm at level C4-5 (Figures 2, 3). Right VC was reported to be in the paramedian position (consistent with clinical findings).

**Figure 4:**
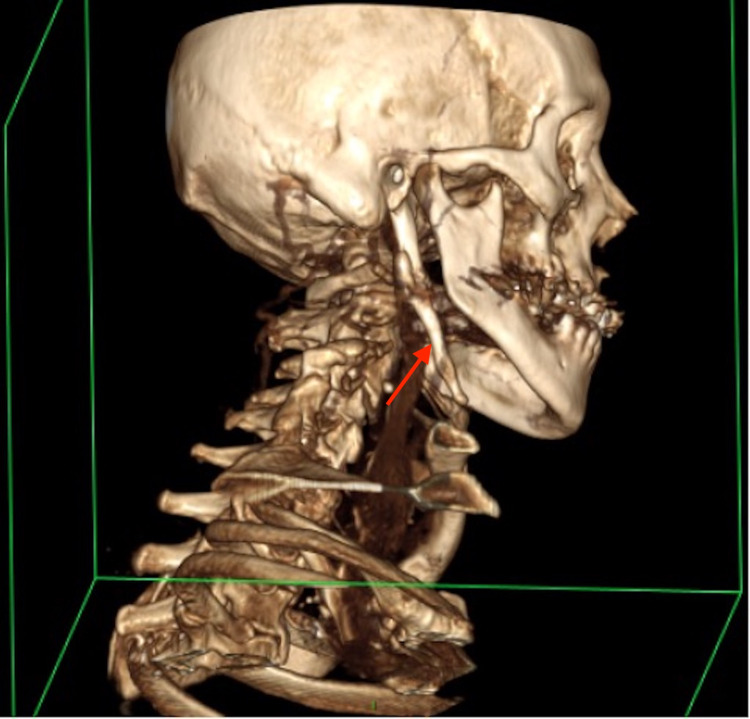
Three-dimensional CT scan of the head and neck showing enlarged, thickened right styloid process with complete ossification of the stylohyoid ligament, and significant bony overgrowth of the pseudoarticulation between the tip of the styloid process and the ossified ligament.

**Figure 5:**
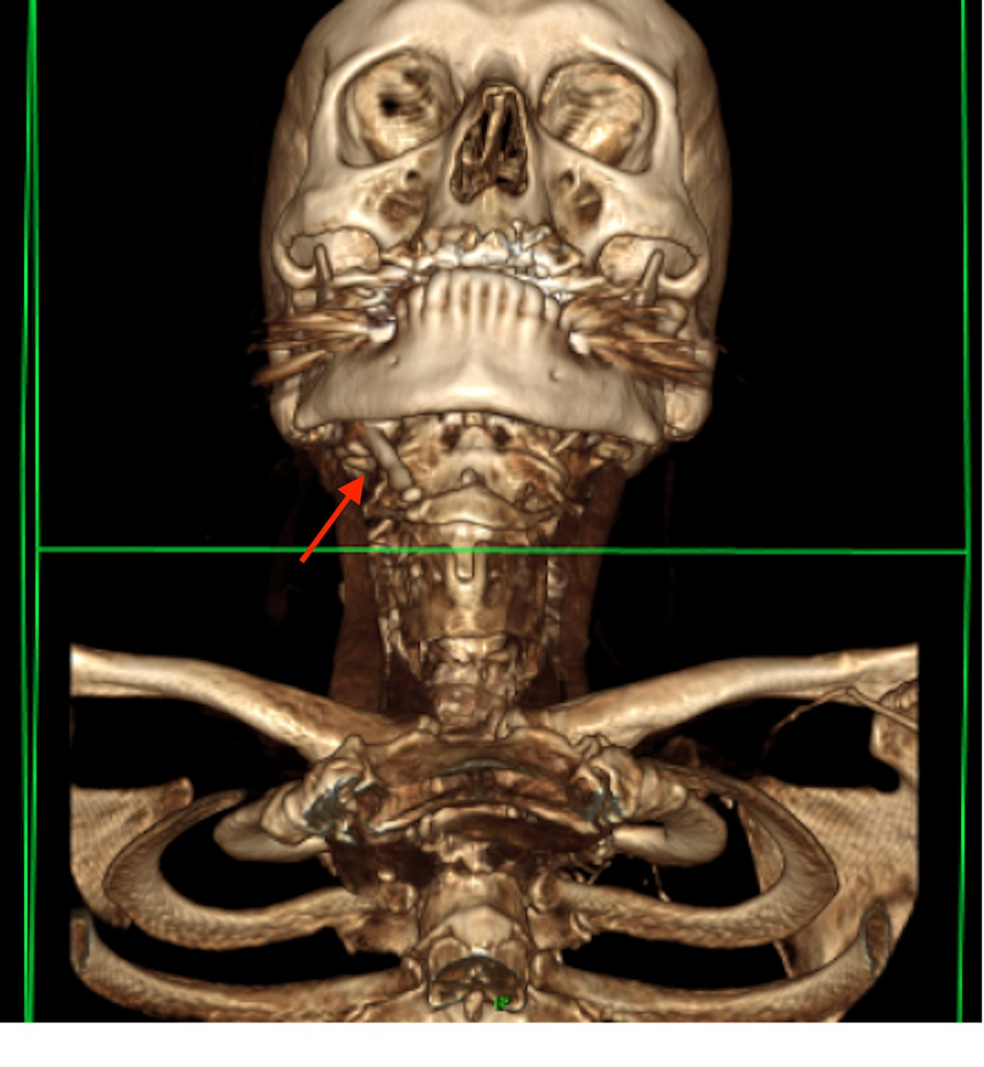
Three-dimensional CT scan of the head and neck showing enlarged, thickened right styloid process with complete ossification of the stylohyoid ligament, and significant bony overgrowth of the pseudoarticulation between the tip of the styloid process and the ossified ligament.

The patient returned to the ENT clinic three years later, complaining of worsening pain in the right submandibular region, and was keen to discuss surgical management. An updated cross-sectional imaging (three-dimensional CT scan) revealed an enlarged, thickened right styloid process with complete ossification of the stylohyoid ligament, and significant bony overgrowth of the pseudoarticulation between the tip of the styloid process and the ossified ligament (Figures 4, 5). The jugular vein was noted inferolaterally to the jugular foramen, passing posterolaterally to the styloid process and the pseudoarticulation with mild potential compression. Although the vagus nerve was not visualised, given it ought to be situated medial to the vein, there was an increased likelihood of its impingement by the bony abnormality. The scan also noted significant spondylotic changes in C4-T1 vertebrae.

As the patient reported significantly reduced quality of life due to pain, the conservative management option was no longer satisfactory. A surgical intervention was proposed - partial styloidectomy and VC injection - for VC paralysis secondary to impingement on the vagal nerve by the stylohyoid complex. The patient underwent this procedure with removal of the offending stylohyoid apparatus (sample sent to pathology) and excision of the stylohyoid muscle, which was found to be completely calcified between the hyoid and styloid process. A large fracture was noted within the calcification at the styloid process junction. There were a few challenges encountered intra-operatively. Firstly, the bony styloid process was attached to the hyoid bone and it was difficult to disarticulate the two, as hypoglossal nerve runs in close proximity to the greater cornua of the hyoid bone. The hyperostotic bony lesion at the proximal end of the styloid process obscured the skull base view, making the identification of jugular foramen contents extremely challenging. Drilling the hyperostosis was limited due to its medial relation to the internal jugular vein to avoid haemorrhage at the skull base and damage to the lower cranial nerves. The anatomical structures were displaced, and identification of the facial nerve close to the stylomastoid foramen/tympanomastoid suture was difficult due to the spherical nature of the bony overgrowth. The neurovascular structures were successfully preserved by careful dissection. Bony attachment was rounded off using a drill superiorly and medially. Polyvinylpyrrolidone was injected to the right VC in order to improve the symptoms of vagus nerve palsy.

The patient recovered well post-operatively and denied any recurrence of stylalgia during the follow-up appointment two months later.

## Discussion

Given the non-specific symptoms, there is a broad list of differential diagnoses that need to be taken into consideration when assessing patients presenting with cervicofacial pain. It is important to exclude trigeminal, sphenopalatine and glossopharyngeal neuralgias, temporo-manidular join dysfunction, naso/oropharyngeal and tongue base tumours, temporal arteritis, salivary gland disease, carotidynia or chronic tonsillitis, chronic laryngopharyngeal reflux and many more. Eagle’s syndrome is generally diagnosed following a combination of clinical examination and imaging. Clinical examination includes palpation of the tonsillar fossa or injecting local anaesthetic into the tonsillar fossa with the aim of inducing pain relief. Three-dimensional CT is the imaging modality of choice and aims to define the length, angulation and anatomy of the abnormal stylohyoid complex [1,2]. In our patient's case, the preoperative CT scan revealed a thickened right styloid process, complete ossification of the stylohyoid ligament and a significant bony overgrowth around the pseudoarticulation present between the tip of the styloid process and the ossified ligament. The extent of the progression of the ossification of the stylohyoid complex did pose significant operative challenges.

Eagle’s syndrome was first described in 1937 as orocervicofacial pain secondary to elongation of the styloid process, hyoid bone or ossification of the stylohyoid ligament [1]. Two syndromes were initially described: classic styloid syndrome and carotid artery or stylocarotid syndrome. Classic styloid syndrome, presented with sore throat, pain at tonsillar fossa and foreign body sensation in the pharynx. This could arise secondary to tonsillectomy due to post-traumatic scarring and hyperplasia [2]. Carotid artery syndrome or stylocarotid syndrome occurs as a result of compression of the stylohyoid complex on the surrounding neurovascular structures, generally presenting with lateral neck pain [1,2].

SHCS is a more recent nomenclature encompassing pathologies associated with various components of the complex, which includes the stylohyoid process, stylohyoid ligament and lesser cornu of the hyoid bone [1-7].

The prevalence of stylohyoid complex pathology was estimated by Gossman and Tristano at approximately 4% of the population, more commonly seen in females, with mean age between 30 and 50 years [4,8]. Eagle's syndrome can be idiopathic, congenital or acquired. Symptoms can be triggered by granulation tissue, secondary to fracture, applying pressure on surrounding structures. This includes impingement on the adjacent neurovascular structures (external and internal carotid arteries, glossopharyngeal nerve, lower branch of trigeminal nerve and chorda tympani), insertion tendinitis (due to degenerative and inflammatory changes) or irritation of the pharyngeal mucosa secondary to post-tonsillectomy scarring [2].

Patients can be managed conservatively or with surgery. Conservative management includes non-steroidal anti-inflammatory drugs, steroids, repeated local anaesthetic injections and neck exercises if the patient is amenable [4]. Surgical management focuses on removal of the offending apparatus by styloidectomy or shortening of the styloid process. A transoral or cervical approach can be taken, and the decision is based on the length of the process, extent of ossification and anatomical relationships. Traditionally, tonsillectomy was performed with the transoral approach; however, tonsil-sparing surgery was also noted to be an effective treatment [4].

Bensoussan et al. described the first Eagle’s syndrome case causing motor paralysis of a cranial nerve in a patient with ipsilateral Horner’s syndrome and hypoglossal nerve palsy [5]. Facial nerve palsy was also described secondary to the elongated styloid syndrome [6]. It was suggested that repeated microinjuries and compression of the neurovascular structures can cause neuropraxia and axonotmesis [4,5]. Given the proximity of the vagal nerve to the stylohyoid complex, impingement on the nerve by the highly ossified ligament and elongated styloid process can be expected. However, to our knowledge, this is the first case of VC palsy associated with Eagle’s syndrome.

As noted previously, the patient had initially opted for conservative management. However, it is possible that an earlier surgical management may have prevented the complication of VC palsy. Due to progression of hyperostosis, the surgical management was very challenging and symptoms of vagus nerve paralysis improved mildly; hence, earlier surgical intervention could have resulted in better outcomes.

## Conclusions

A case of right VC palsy secondary to vagal nerve compression by an elongated right styloid process and calcified stylohyoid complex was noted. Although conservative management of Eagle’s syndrome can prove effective for some patients, we would advise close monitoring of these cases. Delayed surgical intervention can lead to the complete ossification of the stylohyoid complex, making it very difficult to proceed with the transoral approach and causing associated symptoms due to compression on neurovascular structures. It is important to be mindful that Eagle’s syndrome can be a cause of cervicofacial pain and may present with sensory or motor dysfunction of the cranial nerves.
